# Association between neutrophil percentage-to-albumin ratio and bone mineral density and prevalent osteoporosis in patients with type 2 diabetes mellitus

**DOI:** 10.3389/fendo.2026.1872715

**Published:** 2026-06-29

**Authors:** Qimin Gao, Nuo Jiang, Xinxin Liu, Xiaoli Wang

**Affiliations:** 1 Tongde Hospital of Zhejiang Province Affiliated to Zhejiang Chinese Medical University (College of Integrated Traditional Chinese and Western Medicine Clinical Medicine), Hangzhou, China; 2 Electromyography Room, Department of Functional Examination, Tongde Hospital ofZhejiang Province, Hangzhou, China

**Keywords:** inflammation, low bone mass, neutrophil percentage-to-albumin ratio, osteoporosis, type 2 diabetes mellitus

## Abstract

**Background:**

Patients with type 2 diabetes mellitus (T2DM) have an increased risk of osteoporosis and fragility fractures. Chronic low-grade inflammation and nutritional imbalance are key underlying mechanisms. The neutrophil percentage-to-albumin ratio (NPAR) is a novel biomarker reflecting both processes, but its role in osteoporosis remains unclear. This study aimed to investigate the association between NPAR and bone mineral density (BMD), as well as prevalent osteoporosis, in patients with T2DM.

**Methods:**

This retrospective cross-sectional study included 259 patients with T2DM. Osteoporosis was diagnosed according to the World Health Organization (WHO) criteria based on dual-energy X-ray absorptiometry results. Logistic regression was used to assess the association between NPAR andprevalent osteoporosis. Linear regression evaluated its relationship with BMD at the femoral neck and total hip. Receiver operating characteristic analysis assessed its concurrent discriminative capacity for prevalent osteoporosis.

**Results:**

NPAR levels were higher in the osteoporosis group (P = 0.004). NPAR was independently associated with osteoporosis (OR = 3.42, 95% CI: 1.10-10.64, P = 0.034). It was negatively associated with femoral neck BMD (b = -0.08, 95% CI: -0.14 to -0.02, P = 0.009) and total hip BMD (b = -0.08, 95% CI: -0.14 to -0.01, P = 0.020). The AUC was 0.62 (95% CI: 0.54-0.71, P = 0.004).

**Conclusion:**

Given its modest concurrent discriminative ability, NPAR should be interpreted in combination with other clinical indicators rather than used as an independent screening marker for disease diagnosis, but may provide supplementary information regarding prevalent osteoporosis in patients with T2DM.

## 1 Introduction

With global population aging and the rising burden of metabolic diseases, the coexistence of type 2 diabetes mellitus (T2DM) and osteoporosis has become increasingly common and represents a major public health challenge (1, 2). Epidemiological data indicate that the global number of patients with diabetes has exceeded 537 million, of whom approximately 90% have T2DM (3). In China, the prevalence of diabetes among adults has exceeded 10%, indicating a continuously increasing disease burden (4). Osteoporosis, a chronic disease closely related to population aging, is also increasing in prevalence. Osteoporotic fractures occur frequently in middle-aged and elderly populations (5). Notably, patients with T2DM have a 2–6-fold higher risk of fractures compared with non-diabetic individuals (6). Although bone mineral density (BMD) in patients with T2DM is often normal or even elevated, fracture risk is significantly increased. This suggests a distinct pathophysiological mechanism of diabetic bone disease, which may involve reduced bone quality and impaired bone microarchitecture (7–9). Therefore, identifying clinical factors associated with low bone mass or osteoporosis in patients with T2DM has become an important focus of clinical research.

In recent years, chronic low-grade inflammation has been recognized as a key link between T2DM and abnormal bone metabolism. Inflammatory responses promote osteoclast differentiation and activation, while inhibiting osteoblast function. This accelerates bone loss (10, 11). In addition, nutritional status plays a crucial role in bone metabolism. Inadequate protein intake or malnutrition can directly impair bone formation and exacerbate bone loss. Previous studies have demonstrated that low body weight, hypoalbuminemia, and reduced prealbumin levels are closely associated with osteoporosis in patients with T2DM (12, 13). Therefore, composite indicators that simultaneously reflect inflammatory status and nutritional condition may offer greater clinical value in assessing prevalent osteoporosis status.

Previous studies have mainly focused on single inflammatory markers in relation to BMD or fracture risk (14). The neutrophil percentage-to-albumin ratio (NPAR) is a composite biomarker integrating inflammatory and nutritional status. It is calculated as the ratio of neutrophil percentage to serum albumin (15). Neutrophil percentage reflects systemic inflammation, whereas serum albumin reflects nutritional status and is also linked to chronic inflammation. Hypoalbuminemia has been associated with an increased risk of osteoporosis (12). Therefore, NPAR may provide a more comprehensive assessment of bone metabolism by capturing both processes.

However, evidence on the association between NPAR and BMD or prevalent osteoporosis in patients with T2DM remains limited. This study aimed to investigate the association between NPAR, BMD, and prevalent osteoporosis in a cohort of 259 patients with T2DM.

## 2 Materials and methods

### 2.1 Study design and population

This was a single-center retrospective cross-sectional study. A total of 359 hospitalized patients admitted to the Department of Endocrinology at Zhejiang Provincial Tongde Hospital between June and October 2025 were initially screened. The inclusion criteria were as follows: (1) diagnosis of T2DM according to the American Diabetes Association (ADA) criteria (16); (2) postmenopausal women or men aged ≥ 50 years; (3) availability of complete clinical data, including neutrophil percentage, serum albumin, and BMD measurements.

**Figure 1:**
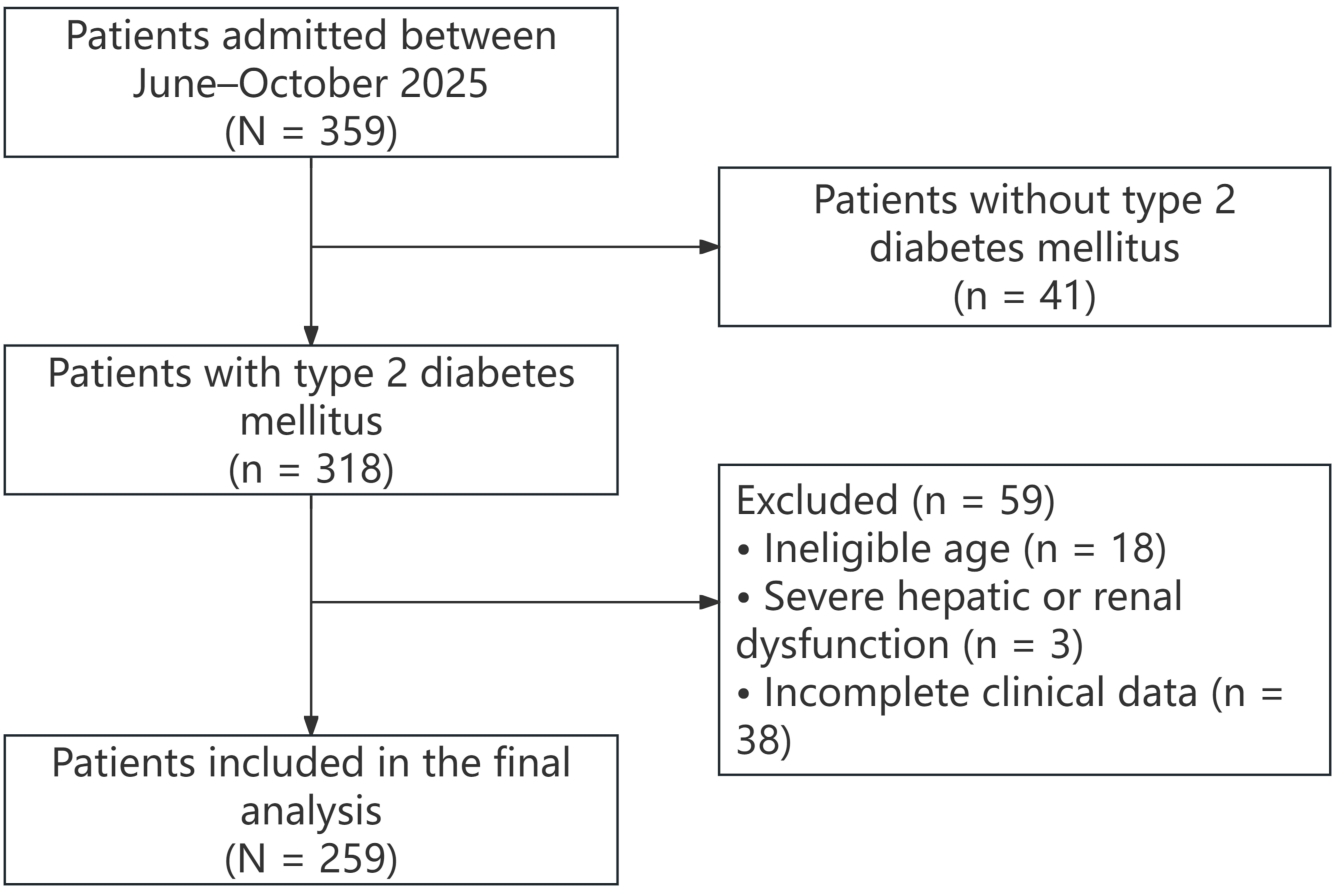
Flowchart of participant selection.

The exclusion criteria were as follows: (1) type 1 diabetes mellitus, gestational diabetes mellitus, or other specific types of diabetes; (2) malignancy, severe hepatic or renal dysfunction (estimated glomerular filtration rate < 30 mL/min/1.73 m²), severe infection, or acute stress conditions; (3) use of medications affecting bone metabolism (e.g., glucocorticoids, bisphosphonates) within the preceding 3 months; (4) pregnancy or lactation. After applying the inclusion and exclusion criteria, 259 patients were included in the final analysis. The patient selection process is shown in **Figure 1**. The study was performed in line with the ethical principles outlined in the Declaration of Helsinki and obtained ethical clearance from the Ethics Committee of Zhejiang Provincial Tongde Hospital (Approval No. ZTD-2025-395-JY). Given the study’s retrospective design, the require ment for written informed consent was waived.

### 2.2 Data collection

Clinical data were extracted from the hospital electronic medical record system, including: (1) Demographic characteristics: age, sex, height, weight, and body mass index (BMI); (2) Clinical variables: duration of diabetes, smoking history, alcohol consumption, systolic blood pressure (SBP), and diastolic blood pressure (DBP); (3) Laboratory parameters: HbA1c; lipid profile, including total cholesterol (TC), triglycerides (TG), low-density lipoprotein cholesterol (LDL-C), and high-density lipoprotein cholesterol (HDL-C); liver function indices [alanine aminotransferase (ALT), aspartate aminotransferase (AST)]; renal function [estimated glomerular filtration rate (eGFR)]; serum calcium; white blood cell count (WBC); absolute neutrophil count (ANC); neutrophil percentage; and serum albumin; (4) NPAR calculation: NPAR was calculated as neutrophil percentage (%) divided by serum albumin (g/L); (5) BMI calculation: BMI was calculated as weight (kg) divided by height squared (m²).

### 2.3 BMD measurement and grouping

BMD and T-scores were measured at the lumbar spine, femoral neck, and total hip using dual-energy X-ray absorptiometry (DXA; GE Lunar Prodigy). According to the World Health Organization (WHO) criteria and the Royal Australian College of General Practitioners (RACGP) 2024 guidelines (17, 18), for postmenopausal women and men aged ≥ 50 years, osteoporosis was defined as a lowest T-score of ≤ -2.5 at any of the measured sites (lumbar spine, femoral neck, or total hip). Normal bone mass was defined as T-score ≥ -1.0, and osteopenia as -2.5 < T-score < -1.0.

In this study, individuals with normal bone mass and those with osteopenia were combined into a single non-osteoporosis group because the study objective was to investigate the association between NPAR and osteoporosis status, not to distinguish subtle differences between normal bone mass and osteopenia.

### 2.4 Statistical analysis

Statistical analyses were performed using SPSS version 27.0 and R software version 4.5.2. The distribution of continuous variables was assessed using the Shapiro-Wilk test. Normally distributed variables are presented as mean ± standard deviation (SD) and were compared using the independent-samples *t*-test. Non-normally distributed variables are presented as median (interquartile range) and were compared using the Mann-Whitney U test. Categorical variables are presented as counts (%) and were compared using the chi-square test.

Univariate and multivariable logistic regression analyses were performed to evaluate the association between NPAR and prevalent osteoporosis. Results are reported as odds ratios (ORs) with 95% confidence intervals (CIs). Linear regression analyses were used to examine the associations between NPAR and BMD at the femoral neck and total hip. Receiver operating characteristic (ROC) curve analysis was performed to evaluate the discriminative performance of NPAR for prevalent osteoporosis. The area under the curve (AUC) and corresponding 95% CIs were calculated. Differences between the AUCs were compared using the DeLong test. Three regression models were constructed. Model 1 was unadjusted. Model 2 was adjusted for age, sex, and BMI. Model 3 was further adjusted for diabetes duration and HDL-C. Covariates included in the multivariable models were selected based on clinical relevance, findings from the univariate analysis, and their potential confounding effects. Given the limited number of osteoporosis events in the study population, the number of covariates included in the fully adjusted model was restricted to reduce the risk of model overfitting. To evaluate NPAR beyond conventional clinical factors, integrated discrimination improvement (IDI) and continuous net reclassification improvement (NRI) were calculated as exploratory assessments after adding NPAR to a baseline model comprising age, sex, and BMI. Corresponding 95% confidence intervals were estimated.

Multicollinearity was assessed using the variance inflation factor (VIF), and a VIF value > 5 was considered indicative of significant multicollinearity. Model calibration was evaluated using the Hosmer–Lemeshow goodness-of-fit test. All statistical tests were two-sided. *P* value < 0.05 was considered statistically significant.

## 3 Results

### 3.1 Characteristics of study participants

A total of 259 patients with T2DM were included, comprising 59 patients (22.8%) in the osteoporosis group and 200 patients (77.2%) in the non-osteoporosis group. Compared with the non-osteoporosis group, patients in the osteoporosis group were older, had a higher proportion of females, lower BMI, higher SBP, longer duration of diabetes, higher neutrophil percentage, and higher NPAR levels (all *P* < 0.05). In addition, serum calcium, ALT, TG, and HDL-C differed significantly between the two groups (all *P* < 0.05). No significant differences were observed in DBP, smoking status, alcohol consumption, HbA1c, eGFR, AST, TC, LDL-C, WBC, ANC, or albumin levels (all *P* > 0.05) (**Table 1**).

**Table 1:** Characteristics of study participants.

Variables	Total (N = 259)	Non-osteoporosis (n=200)	Osteoporosis (n=59)	*P* value
Age (years)	64.00 (55.00, 72.00)	62.00 (53.50, 70.00)	69.00 (59.00, 75.00)	**0.001**
Female, n (%)	97 (37.5%)	60 (30.0%)	37 (62.7%)	**<0.001**
BMI (kg/m2)	23.84 (21.67, 25.91)	24.42 (22.52, 26.31)	22.06 (20.37, 23.50)	**<0.001**
SBP (mmHg)	135.00 (123.00, 145.00)	133.00 (121.50, 145.00)	139.00 (127.50, 149.50)	**0.032**
DBP (mmHg)	82.21 ± 10.63	82.50 ± 10.39	81.24 ± 11.45	0.426
Smoking, n (%)	42 (16.2%)	36 (18.0%)	6 (10.2%)	0.152
Alcohol consumption, n (%)	28 (10.8%)	23 (11.5%)	5 (8.5%)	0.511
Duration of diabetes (years)	6.00 (2.50, 15.00)	5.00 (2.00, 10.50)	10.00 (4.50, 20.00)	**0.004**
HbA1c (%)	9.60 (7.80, 11.20)	9.60 (7.80, 11.20)	9.50 (7.75, 11.60)	0.944
eGFR (mL/min/1.73m²)	93.32 (77.51, 103.00)	93.89 (79.74, 103.25)	92.81 (75.00, 102.00)	0.581
ALT (U/L)	21.00 (16.00, 32.00)	22.00 (17.00, 34.00)	19.00 (13.50, 28.00)	**0.016**
AST (U/L)	22.00 (18.00, 29.00)	22.00 (18.00, 29.00)	21.00 (18.00, 25.50)	0.346
TC (mmol/L)	4.78 ± 1.32	4.72 ± 1.35	5.00 ± 1.20	0.144
TG (mmol/L)	1.42 (0.99, 2.04)	1.47 (1.05, 2.09)	1.21 (0.82, 1.83)	**0.045**
HDL-C (mmol/L)	1.17 (1.00, 1.40)	1.14 (0.97, 1.36)	1.31 (1.15, 1.65)	**<0.001**
LDL-C (mmol/L)	2.84 (2.25, 3.58)	2.83 (2.22, 3.58)	2.91 (2.39, 3.61)	0.470
Serum calcium (mmol/L)	2.32 (2.23, 2.40)	2.33 (2.24, 2.40)	2.26 (2.20, 2.35)	**0.008**
WBC (*10^9^/L)	5.80 (4.99, 7.00)	5.93 (5.07, 7.13)	5.65 (4.62, 6.84)	0.161
ANC (*10^9^/L)	3.60 (2.90, 4.50)	3.60 (2.90, 4.60)	3.70 (3.00, 4.40)	0.945
Neutrophil percentage (%)	61.80 (55.30, 68.80)	61.05 (54.80, 67.4)	65.30 (58.70, 74.2)	**0.013**
Albumin (g/L)	41.07 ± 4.40	41.33 ± 4.41	40.20 ± 4.31	0.085
**NPAR**	1.52 (1.31, 1.73)	1.49 (1.29, 1.67)	1.62 (1.35, 1.81)	**0.004**

Data are presented as mean ± SD, median (IQR), or n (%). Normally distributed continuous variables were compared using the independent samples t-test; non-normally distributed variables were compared using the Mann–Whitney U test. Categorical variables were compared using the chi-square (χ²) test. Bold values indicate statistically significant differences at *P* < 0.05.

BMI, body mass index; SBP, systolic blood pressure; DBP, diastolic blood pressure; HbA1c, glycated hemoglobin; eGFR, estimated glomerular filtration rate; ALT, alanine aminotransferase; AST, aspartate aminotransferase; TC, total cholesterol; TG, triglycerides; HDL-C, high-density lipoprotein cholesterol; LDL-C, low-density lipoprotein cholesterol; WBC, white blood cell count; ANC, absolute neutrophil count; NPAR, neutrophil percentage-to-albumin ratio.

### 3.2 Univariate logistic regression analysis of factors associated with osteoporosis

Univariate logistic regression analysis showed that NPAR was positively associated with osteoporosis (OR = 4.21, 95% CI: 1.61-10.99, *P* = 0.003). Age (OR = 1.05, 95% CI: 1.02-1.08, *P* = 0.001), female sex (OR = 3.92, 95% CI: 2.14-7.21, *P* < 0.001), duration of diabetes (OR = 1.06, 95% CI: 1.02-1.09, *P* = 0.002), HDL-C (OR = 5.26, 95% CI: 2.16-12.82, *P* < 0.001), and neutrophil percentage (OR = 1.04, 95% CI: 1.01-1.07, *P* = 0.018) were positively associated with osteoporosis. In contrast, BMI was inversely associated with osteoporosis (OR = 0.73, 95% CI: 0.64-0.82, *P* < 0.001). No significant associations were observed for the remaining variables (all *P* > 0.05) (**Table 2**).

**Table 2:** Univariate logistic regression analysis of factors associated with osteoporosis.

Variables	OR (95% CI)	*P* value
Age (year)	1.05 (1.02-1.08)	**0.001**
Female (vs. male)	3.92 (2.14-7.21)	**<0.001**
BMI (kg/m2)	0.73 (0.64-0.82)	**<0.001**
SBP (mmHg)	1.02 (1.00-1.03)	0.076
DBP (mmHg)	0.99 (0.96-1.02)	0.424
Duration of diabetes (years)	1.06 (1.02-1.09)	**0.002**
HbA1c (%)	1.02 (0.90-1.14)	0.784
TC (mmol/L)	1.18 (0.95-1.47)	0.145
TG (mmol/L)	0.82 (0.62-1.08)	0.151
LDL-C (mmol/L)	1.12 (0.82-1.53)	0.490
HDL-C (mmol/L)	5.26 (2.16-12.82)	**<0.001**
eGFR (mL/min/1.73m²)	0.99 (0.98-1.01)	0.395
ALT (U/L)	0.98 (0.96-1.00)	0.102
AST (U/L)	0.99 (0.96-1.02)	0.351
Serum calcium (mmol/L)	1.36 (0.62-2.96)	0.442
WBC (*10^9^/L)	0.86 (0.70-1.04)	0.126
ANC (*10^9^/L)	0.98 (0.79-1.21)	0.831
Neutrophil percentage (%)	1.04 (1.01-1.07)	**0.018**
Albumin (g/L)	0.94 (0.88-1.01)	0.087
NPAR	4.21 (1.61-10.99)	**0.003**

Data are presented as odds ratio (OR) with 95% confidence interval (CI). Bold values indicate statistically significant differences at *P* < 0.05.

BMI, body mass index; SBP, systolic blood pressure; DBP, diastolic blood pressure; HbA1c, glycated hemoglobin; eGFR, estimated glomerular filtration rate; ALT, alanine aminotransferase; AST, aspartate aminotransferase; TC, total cholesterol; TG, triglycerides; HDL-C, high-density lipoprotein cholesterol; LDL-C, low-density lipoprotein cholesterol; WBC, white blood cell count; ANC, absolute neutrophil count; NPAR, neutrophil percentage-to-albumin ratio.

### 3.3 Multivariable logistic regression analysis of osteoporosis

Multivariate logistic regression analysis demonstrated that the association between NPAR and osteoporosis remained robust after sequential adjustment for potential confounders. In the unadjusted model (Model 1), NPAR was associated with osteoporosis (OR = 4.21, 95% CI: 1.61-10.99, *P* = 0.003). This association remained significant after adjustment for age, sex, and BMI (Model 2: OR = 3.12, 95% CI: 1.04-9.41, *P* = 0.043), and after further adjustment for duration of diabetes and HDL-C (Model 3: OR = 3.42, 95% CI: 1.10-10.64, *P* = 0.034). Female sex and lower BMI remained independently associated with osteoporosis in the adjusted models (**Table 3**). The Hosmer-Lemeshow test indicated a good model fit (χ² = 6.457, df = 8, *P* = 0.596). All VIF values were below 1.5, indicating no significant multicollinearity. To further evaluate the robustness of the findings, sensitivity analyses were performed by additionally adjusting for WBC and ANC, respectively. The association between NPAR and prevalent osteoporosis remained statistically significant after adjustment for WBC (OR = 3.76, 95% CI: 1.17-12.07, *P* = 0.026) and ANC (OR = 4.26, 95% CI: 1.16-15.66, *P* = 0.029), supporting the stability and robustness of the primary findings (**Supplementary Table 1**).

**Table 3:** Multivariable logistic regression analysis of osteoporosis.

Variables	Model 1 OR (95% CI)	*P* value	Model 2 OR (95% CI)	*P* value	Model 3 OR (95% CI)	*P* value
NPAR	4.21 (1.61-10.99)	**0.003**	3.12 (1.04-9.41)	**0.043**	3.42 (1.10-10.64)	**0.034**
Female (vs. male)	/	/	3.57 (1.82-6.97)	**<0.001**	3.21 (1.61-6.37)	**<0.001**
Age (year)	/	/	1.02 (0.99-1.06)	0.206	1.01 (0.97-1.05)	0.555
BMI(kg/m2)	/	/	0.77 (0.68-0.88)	**<0.001**	0.80 (0.71-0.91)	**<0.001**
Duration of diabetes (years)	/	/	/	**/**	1.03 (0.99-1.07)	0.178
HDL-C (mmol/L)	/	/	/	**/**	2.25 (0.82-6.17)	0.116

Model 1: unadjusted. Model 2: adjusted for age, sex, and BMI. Model 3: further adjusted for duration of diabetes and HDL-C.

Data are presented as odds ratio (OR) with 95% confidence interval (CI). Bold values indicate statistically significant differences at *P* < 0.05.

OR, odds ratio; CI, confidence interval; BMI, body mass index; HDL-C, high-density lipoprotein cholesterol.

### 3.4 Linear regression analysis of the association between NPAR and BMD at the femoral neck and total hip

NPAR was negatively associated with BMD at both the femoral neck and total hip. In Model 1, higher NPAR was associated with lower femoral neck BMD (β = -0.11, 95% CI: -0.17 to -0.05, *P* < 0.001) and lower total hip BMD (β = -0.09, 95% CI: -0.15 to -0.02, *P* = 0.014). After adjustment for age, sex, and BMI (Model 2), these associations remained significant for both femoral neck BMD (β = -0.08, 95% CI: -0.13 to -0.02, *P* = 0.011) and total hip BMD (β = -0.07, 95% CI: -0.14 to -0.01, *P* = 0.027). Further adjustment for duration of diabetes and HDL-C (Model 3) did not materially alter the results. NPAR remained independently associated with femoral neck BMD (β = -0.08, 95% CI: -0.14 to -0.02, *P* = 0.009) and total hip BMD (β = -0.08, 95% CI: -0.14 to -0.01, *P* = 0.020) (**Table 4**).

**Table 4:** Linear regression analysis of the association between NPAR and BMD at the femoral neck and total hip.

Outcome	Model 1 β(95% CI)	*P* value	Model 2 β(95% CI)	*P* value	Model 3 β(95% CI)	*P* value
Femoral neck BMD	-0.11 (-0.17 to -0.05)	**<0.001**	-0.08 (-0.13 to -0.02)	**0.011**	-0.08 (-0.14 to -0.02)	**0.009**
Total hip BMD	-0.09 (-0.15 to -0.02)	**0.014**	-0.07 (-0.14 to -0.01)	**0.027**	-0.08 (-0.14 to -0.01)	**0.020**

Model 1: unadjusted. Model 2: adjusted for age, sex, and BMI. Model 3: further adjusted for duration of diabetes and HDL-C.

Data are presented as β coefficients (β) with 95% confidence intervals (CI). Bold values indicate statistically significant differences at *P* < 0.05.

BMD, bone mineral density; CI, confidence interval; BMI, body mass index; HDL-C, high-density lipoprotein cholesterol.

### 3.5 ROC curve analysis

**Figure 2:**
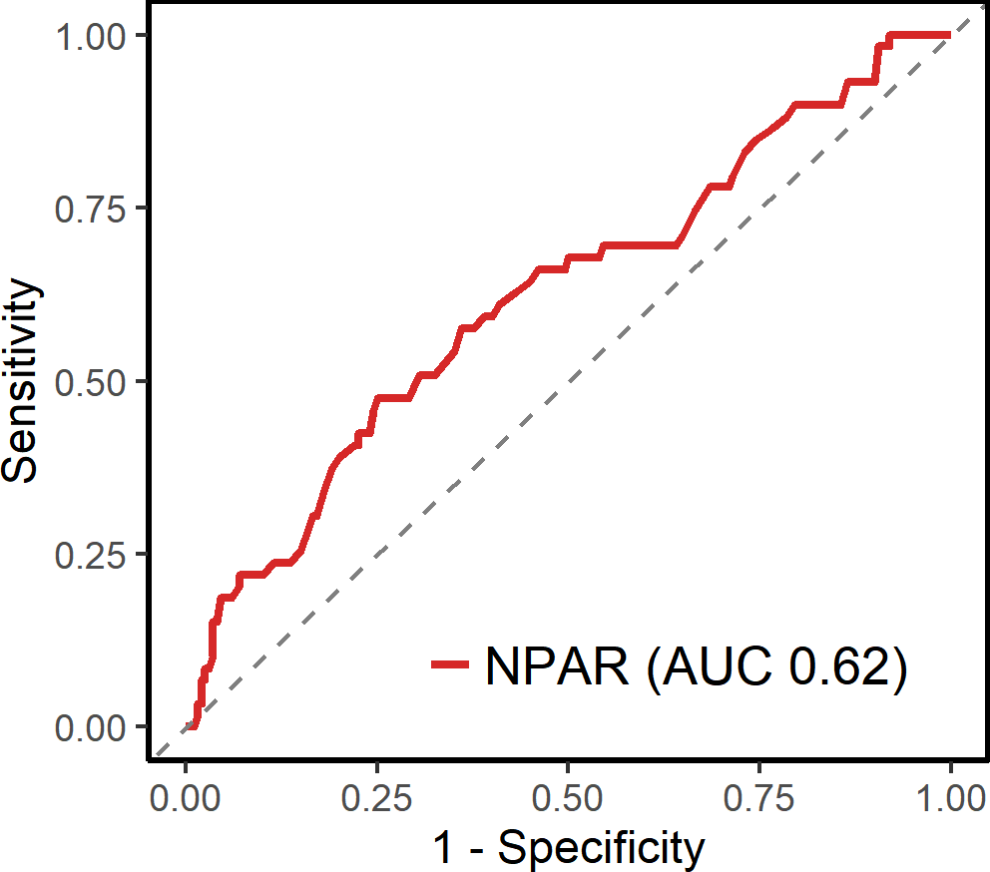
ROC curve of NPAR for discriminating prevalent osteoporosis in patients with T2DM. The area under the curve (AUC) was 0.62 (95% CI: 0.54-0.71, *P* = 0.004).

ROC curve analysis showed that NPAR had a modest ability to discriminate osteoporosis. The AUC was 0.62 (95% CI: 0.54-0.71, *P* = 0.004). The optimal cut-off value was 1.665, with a sensitivity of 47.5% and a specificity of 75.0% (**Figure 2**). We further compared the discriminative performance of NPAR with its individual components (**Supplementary Table 2**). Although NPAR exhibited a numerically higher AUC (0.62) compared to neutrophil percentage (0.61) and albumin (0.57) alone, DeLong tests indicated that these differences were not statistically significant (both *P^a^* > 0.05).

### 3.6 Exploratory assessment of NPAR beyond conventional clinical factors

To test whether NPAR improves discrimination beyond the baseline model (comprising age, sex, and BMI), we compared their predictive performances. The addition of NPAR to the baseline model did not result in a statistically significant improvement in the AUC. Similarly, the resulting IDI was 0.017 (95% CI: -0.003 to 0.035, *P* = 0.086), which was not statistically significant. Although the continuous NRI showed a statistically significant value of 0.323 (95% CI: 0.030 to 0.584, *P* = 0.036), given the non-significant AUC improvement and IDI, this continuous NRI result is reported strictly as an exploratory finding (**Table 5**).

**Table 5:** Exploratory assessment of NPAR beyond conventional clinical factors.

Metric	Estimate (95% CI)	*P* value
IDI	0.017 (-0.003 to 0.035)	0.086
NRI	0.323 (0.030 to 0.584)	**0.036**

Baseline model included age, sex, and BMI.

Exploratory analyses were performed by adding NPAR to the baseline model.

IDI, Integrated Discrimination Improvement; NRI, Net Reclassification Improvement.

Estimate refers to the IDI value for the IDI row and the NRI value for the NRI row. The original sentence is replaced with: Bold values indicate statistically significant differences at *P* < 0.05.

## 4 Discussion

In this cross-sectional study of 259 patients with T2DM, NPAR levels were significantly higher in individuals with osteoporosis. NPAR was independently associated with prevalent osteoporosis (OR = 3.42, 95% CI: 1.10-10.64, *P* = 0.034). In addition, NPAR was negatively correlated with BMD at the femoral neckand total hip. Although its discriminative ability was modest (AUC = 0.62), these cross-sectional, retrospective findings consistently suggest that NPAR is associated with prevalent osteoporosis in patients with T2DM.

NPAR, calculated as the ratio of neutrophil percentage to serum albumin, has recently emerged as a novel biomarker integrating inflammatory and nutritional status. Its prognostic value has been increasingly recognized across a range of chronic diseases. Previous studies have demonstrated that NPAR is closely associated with diabetic nephropathy, cardiovascular mortality, and all-cause mortality (19–26). These findings collectively suggest that systemic inflammation and malnutrition play critical roles in the pathophysiology of T2DM. NPAR, by reflecting the state of “inflammation–nutrition imbalance,” may indirectly contribute to impaired bone health.Emerging evidence indicates that chronic low-grade inflammation is one of the key mechanisms underlying impaired bone quality in patients with T2DM (27). Cheng comprehensively described the complex role of neutrophils in bone metabolism, demonstrating that neutrophils may exacerbate bone loss through multiple mechanisms. Specifically, neutrophils can promote osteoclast differentiation and bone resorption via receptor activator of nuclear factor-κB ligand (RANKL) signaling, reactive oxygen species (ROS), and neutrophil extracellular traps (NETs). In addition, they may inhibit osteoblast function either through the secretion of inhibitory cytokines, such as transforming growth factor-β1 (TGF-β1), or via direct cellular interactions (28). In a hyperglycemic environment, neutrophils overproduce 4-guanidinobutyric acid (4-GBA), a metabolite that directly binds to and inhibits alkaline phosphatase (ALP) activity on the surface of bone marrow-derived mesenchymal stem cells (BMSCs), thereby severely impairing their osteogenic differentiation capacity and leading to impaired bone regeneration (29).

Nutritional status also plays a critical role in bone metabolism. Hypoalbuminemia is often accompanied by elevated pro-inflammatory cytokines and has been linked to various metabolic disorders, including malignancy, nephrotic syndrome, and the inflammation–malnutrition syndrome (30, 31). In patients with T2DM, low serum albumin has been identified as an independent risk factor for osteoporosis (32). Albumin exerts multiple physiological functions, including antioxidant activity, endotoxin binding, maintenance of colloid osmotic pressure, and transport of bioactive substances. Reduced albumin levels may contribute to a vicious cycle of inflammation, hypoalbuminemia, and immune dysfunction (33). Moreover, chronic hypoalbuminemia may impair bone formation by reducing collagen synthesis and decreasing the bioavailability of insulin-like growth factor-1 (IGF-1), a key determinant of bone strength (34). Therefore, an elevated NPAR may reflect the combined detrimental effects of systemic inflammation and poor nutritional status on bone metabolism, ultimately contributing to bone loss in patients with T2DM.

Recent studies have begun to explore the relationship between inflammatory markers and BMD or osteoporosis. For instance, a study in postmenopausal women with T2DM reported that inflammatory indices such as the neutrophil-to-lymphocyte ratio (NLR) and monocyte-to-lymphocyte ratio (MLR) were negatively associated with BMD (35). Similarly, another study demonstrated that NLR was associated with osteoporosis risk in non-diabetic postmenopausal women (36). Although these studies did not directly investigate NPAR, their findings support the central role of neutrophil-driven inflammation in the pathogenesis of bone metabolism disorders. As an emerging composite biomarker reflecting both inflammatory and nutritional status, NPAR has recently attracted increasing attention due to its prognostic value in various diseases. However, evidence regarding its association with osteoporosis in patients with T2DM remains scarce. This highlights the novelty and clinical relevance of the present study.A recent analysis based on the National Health and Nutrition Examination Survey (NHANES) reported an association between NPAR and BMD; however, the results were inconsistent after adjustment for multiple confounding factors (37). In contrast, our study demonstrated that NPAR was independently associated with both prevalent osteoporosis and lower BMD in patients with T2DM after multivariable adjustment. Several differences between our study and the NHANES analysis are noteworthy. The NHANES study found that the NPAR-OP association was significant only in males, and that obesity and older age potentiated this association. In contrast, our study identified female sex (OR = 3.21) and lower BMI (OR = 0.80) as independent correlates of osteoporosis, while age was not significant (OR = 1.01, *P* = 0.555). These discrepancies may be attributable to differences in study populations and outcome definitions. This further supports the novelty and clinical value of the present study. This discrepancy may be attributable to differences in study populations. Specifically, our study focused exclusively on individuals with T2DM, a population characterized by more pronounced inflammatory and metabolic disturbances, which may amplify the effect of NPAR on bone metabolism. This further supports the novelty and clinical value of the present study. Furthermore, a study focusing on patients with rheumatoid arthritis(RA) reported that the incidence of osteoporosis was significantly higher in the high-NPAR group than in the low-NPAR group (38). NPAR was independently associated with osteoporosis. These findings are highly consistent with our results (AUC = 0.58 and 0.62, respectively). Together, both studies confirm that NPAR is associated with osteoporosis across different chronic inflammatory diseases (RA and T2DM), suggesting that “inflammation-nutrition imbalance” may represent a shared mechanism underlying disease-related bone metabolism disorders.

The pathogenesis of diabetes-related osteoporosis is complex and multifactorial. Chronic hyperglycemia can lead to the accumulation of advanced glycation end products (AGEs) in bone tissue, which disrupt the bone matrix structure and reduce bone strength (39, 40). In addition, both insulin deficiency and insulin resistance may adversely affect bone formation. Furthermore, diabetes-related complications can increase the risk of falls, thereby further elevating fracture risk (41, 42). In the present study, NPAR remained independently associated with prevalent osteoporosis after multivariable adjustment. This finding is consistent with the hypothesis that inflammatory and nutritional disturbances may be involved in diabetes-related bone disease. Notably, after adjusting for confounding factors, female sex remained independently associated with higher odds of prevalent osteoporosis (OR = 3.21, 95% CI: 1.61-6.37, P < 0.001), and higher BMI with lower odds (OR = 0.80, 95% CI: 0.71-0.91, P < 0.001), consistent with previous findings (43, 44). These associations suggest that female T2DM patients and those with lower BMI deserve particular clinical attention, as they may have a higher likelihood of coexisting osteoporosis. Early clinical awareness of these factors could facilitate timely bone health assessment in this population. We also compared NPAR with its individual components. Although NPAR showed a numerically higher AUC (0.62) than neutrophil percentage (0.61) and serum albumin (0.57) alone, the differences were not statistically significant. This lack of significance is likely due to the inherent correlation between the composite index and its constituents, as well as the limited sample size. Nevertheless, from a pathophysiological perspective, NPAR remains theoretically advantageous by integrating both the systemic inflammation and nutritional pathways into a single index. Furthermore, evaluating NPAR beyond conventional risk factors (age, sex, and BMI) yielded a non-significant IDI. Although the continuous NRI was statistically significant, it is interpreted as an exploratory finding. This reinforces that NPAR should serve merely as an adjunctive reference rather than a primary clinical tool.

From a practical perspective, NPAR offers a non-invasive, cost-effective, and readily accessible biomarker that comprehensively reflects both inflammatory and nutritional status without incurring additional financial or procedural burdens on patients. To our knowledge, this is the first study to explore the independent association between NPAR and bone mineral density status in patients with type 2 diabetes. Given its modest discriminative ability, NPAR should not be used in isolation, but rather in combination with other clinical risk factors as a supplementary reference for identifying high-risk individuals.

Despite these practical advantages, the present study has several limitations that must be acknowledged. First, the cross-sectional design precludes causal inference. Second, although multiple confounders were adjusted for and additional sensitivity analyses yielded similar results, residual confounding cannot be completely excluded. Some potentially relevant variables, including vitamin D, parathyroid hormone, physical activity, and menopausal duration, were not available for all participants due to the retrospective study design and were therefore not included in the analysis. Regarding medication exposure, antidiabetic regimens were highly heterogeneous with frequent changes during the disease course, and only four patients received calcium/vitamin D supplements, with no anti-osteoporosis medication use; therefore, no adjustment was made for these medication variables. Lastly, the relatively small sample size and single-center setting may limit the generalizability of the findings.

## 5 Conclusion

NPAR was independently associated with prevalent osteoporosis and lower femoral neck and total hip BMD in patients with T2DM. Given its limited discriminative ability, NPAR should be used in combination with other clinical indicators as a supplementary reference for osteoporosis status in patients with T2DM. Further prospective studies are needed to clarify its clinical utility.

## Data Availability

The original contributions presented in the study are included in the article/**Supplementary Material**. Further inquiries can be directed to the corresponding author.
