# Supplementary material for: Association between neutrophil percentage-to-albumin ratio and bone mineral density and prevalent osteoporosis in patients with type 2 diabetes mellitus
Source: Front Endocrinol (Lausanne). 2026 Jun 29;17:1872715. doi: 10.3389/fendo.2026.1872715 (PMC13357195; doi:10.3389/fendo.2026.1872715)
Supplement: Supplementary file 1 [file Table1.docx]

**Supplementary Table S1. Sensitivity analyses of the association between NPAR and prevalent osteoporosis after additional adjustment for inflammatory markers.**

| **Model** | **OR(95%CI)** | ***P* value** |
| --- | --- | --- |
| **Model 3** | 3.42 (1.10-10.64) | **0.034** |
| **Model 3 + WBC** | 3.76 (1.17-12.07) | **0.026** |
| **Model 3 + ANC** | 4.26 (1.16-15.66) | **0.029** |

Data are presented as odds ratio (OR) with 95% confidence interval (CI). Bold values indicate statistically significant differences at *P* < 0.05.

Abbreviations: WBC, white blood cell count; ANC, absolute neutrophil count; NPAR,neutrophil percentage-to-albumin ratio.
