# Supplementary material for: Association between neutrophil percentage-to-albumin ratio and bone mineral density and prevalent osteoporosis in patients with type 2 diabetes mellitus
Source: Front Endocrinol (Lausanne). 2026 Jun 29;17:1872715. doi: 10.3389/fendo.2026.1872715 (PMC13357195; doi:10.3389/fendo.2026.1872715)
Supplement: Supplementary file 2 [file Table2.docx]

**Supplementary Table S2. Univariate logistic regression and ROC curve analyses of NPAR, neutrophil percentage, and albumin for osteoporosis risk assessment.**

| **Variable** | **OR (95% CI)** | ***P* value** | **AUC (95% CI)** | ***P* value^a^** |
| --- | --- | --- | --- | --- |
| **NPAR** | 4.21 (1.61-10.99) | **0.003** | 0.62 (0.54-0.71) | Reference |
| **Neutrophil percentage (%)** | 1.04 (1.01-1.07) | **0.018** | 0.61 (0.52-0.69) | 0.463 |
| **Albumin (g/L)** | 0.94 (0.88-1.01) | 0.087 | 0.57 (0.48-0.65) | 0.215 |

Data are presented as odds ratio (OR) with 95% confidence interval (CI) derived from univariate logistic regression analysis. Bold values indicate statistically significant differences at *P* < 0.05.

*P* value^a^ for AUC comparison against NPAR using DeLong's test. NPAR served as the reference category.

Abbreviations: NPAR, neutrophil percentage-to-albumin ratio; AUC, area under the curve.
